# Vaginal Epithelium Transiently Harbours HIV-1 Facilitating Transmission

**DOI:** 10.3389/fcimb.2021.634647

**Published:** 2021-03-17

**Authors:** Varsha M. Prabhu, Varsha Padwal, Shilpa Velhal, Sukeshani Salwe, Vidya Nagar, Priya Patil, Atmaram H. Bandivdekar, Vainav Patel

**Affiliations:** ^1^ Department of Biochemistry and Virology, National Institute for Research in Reproductive Health, Indian Council of Medical Research, Mumbai, India; ^2^ Department of Medicine, The Grant Medical College & Sir J. J. Group of Hospitals, Mumbai, India

**Keywords:** vaginal epithelium, Vk2/E6E7, HIV-1, trans-infection, reservoir, LFA-1, CCR5, α4β7

## Abstract

Vaginal transmission accounts for majority of newly acquired HIV infections worldwide. Initial events that transpire post-viral binding to vaginal epithelium leading to productive infection in the female reproductive tract are not well elucidated. Here, we examined the interaction of HIV-1 with vaginal epithelial cells (VEC) using Vk2/E6E7, an established cell line exhibiting an HIV-binding receptor phenotype (CD4-CCR5-CD206+) similar to primary cells. We observed rapid viral sequestration, as a metabolically active process that was dose-dependent. Sequestered virus demonstrated monophasic decay after 6 hours with a half-life of 22.435 hours, though residual virus was detectable 48 hours’ post-exposure. Viral uptake was not followed by successful reverse transcription and thus productive infection in VEC unlike activated PBMCs. Intraepithelial virus was infectious as evidenced by infection *in trans* of PHA-p stimulated PBMCs on co-culture. Trans-infection efficiency, however, deteriorated with time, concordant with viral retention kinetics, as peak levels of sequestered virus coincided with maximum viral output of co-cultivated PBMCs. Further, blocking lymphocyte receptor function-associated antigen 1 (LFA-1) expressed on PBMCs significantly inhibited trans-infection suggesting that cell-to-cell spread of HIV from epithelium to target cells was LFA-1 mediated. In addition to stimulated PBMCs, we also demonstrated infection *in trans* of FACS sorted CD4+ T lymphocyte subsets expressing co-receptors CCR5 and CXCR4. These included, for the first time, potentially gut homing CD4+ T cell subsets co-expressing integrin α4β7 and CCR5. Our study thus delineates a hitherto unexplored role for the vaginal epithelium as a transient viral reservoir enabling infection of susceptible cell types.

## Introduction

The HIV-1 epidemic currently afflicts 38.0 million individuals worldwide and more than half of people living with HIV (PLHIV) constitute women (UNAIDS, 2020). Mucosal transmission through the genital route, though inefficient (0.08% – 0.3% per act), is the predominant mode of infection in females (Boily et al., 2009). The presence of existing sexually-transmitted diseases can disrupt epithelial integrity and induce local inflammation, greatly increasing the risk of HIV acquisition (Seth, 2011). Even so, in the absence of such facilitatory scenarios, how HIV overcomes several anatomical and physiological barriers of the female reproductive tract (FRT) prior to establishing productive infection remains to be fully understood. Elucidation of a definite mechanism for viral entry and onward transmission to target cells would greatly inform interventions such as PreP, microbicides and vaccines.

Previous studies on non-human primate models have implicated the endocervix (single layer of columnar epithelial cells) and transformation zone (junction between endo- and ectocervix) with its abundance of target cells as principal sites for viral entry in the female reproductive tract (FRT) (Li et al., 2009; Haase, 2010). Conversely, Carias et al demonstrated that the virus cannot efficiently traverse the columnar epithelium due to mucus impediment but can penetrate the intact squamous epithelium of the vagina (Carias et al., 2013). Subsequent studies using more advanced reporting systems in rhesus macaque-SIV vaginal transmission models have shown that infection occurs primarily in the vaginal and ectocervical tissues, highlighting the limitations of cervix-centric models (Stieh et al., 2014; Deleage et al., 2019).

Although, direct infection of genital epithelial cells has been proposed (Asin et al., 2003; Micsenyi et al., 2013), studies spanning tissues of diverse origin suggest that epithelial cells are refractory to infection (Kohli et al., 2014; Yasen et al., 2017). Alternatively, virions may transmigrate the multi-layered epithelium via micro-lacerations which arise during sexual intercourse or by transcytosis, an intracellular trafficking process that utilizes the vesicular/endosomal machinery of the cell (Kinlock et al., 2014; Yasen et al., 2017). Viral transcytosis though, is not always effective as miniscule amounts of the initial inoculum reach the underlying *lamina propria* (Bobardt et al., 2007). It has been proposed that bulk of the virus is sequestered in intracellular compartments of genital epithelial cells which may then disseminate to susceptible cells that populate the squamous epithelium such as macrophages /dendritic cells or intraepithelial CD4+ T cells (Hladik and McElrath, 2008; Yasen et al., 2017; Gonzalez et al., 2019). In a seminal study, Wu et al. showed that ectocervical cells remain uninfected but are capable of transmitting captured virus to CD4+ T lymphocytes (Wu et al., 2003). Numerous studies have since highlighted that CD4+ T lymphocytes are the earliest targets of HIV in the genital epithelium that enable local amplification preceding systemic dissemination (Haase, 2011; Stieh et al., 2014; Deleage et al., 2019).

In a previous study, we have shown the expression of human mannose receptor (hMR) on vaginal epithelial cells as a high affinity receptor that binds HIV-1 Env protein gp120 which in turn induces the production of matrix metalloproteinase-9 (MMP-9), potentially destabilizing the epithelial barrier (Fanibunda et al., 2011). In this study, to elucidate a mechanism for viral transmission through vaginal epithelium to susceptible CD4+ T cell subsets we evaluated interaction of HIV-1 with Vk2/E6E7 cells to delineate a role for vaginal epithelium as a transient viral reservoir permitting onward transmission.

## Materials and Methods

### Primary Cells and Cell Lines 

Clinical samples were obtained from individuals attending ART centre at Sir J.J. Group of Hospitals, Mumbai, with informed consent and approval from the NIRRH Institutional Clinical Ethics Committee (project No. 160/2009 and No. 225/2012). Vaginal epithelial cells were obtained through swab samples collected from women (aged 21-40) with regular menses (28-35 days) during ovulatory phase as described previously (Fanibunda et al., 2011). Peripheral blood was collected from recruited participants for immunophenotyping of lymphocyte and monocyte subsets by flow cytometry.

Human vaginal epithelial cell line Vk2/E6E7, a gift from Dr. Raina Fichorova, Brigham Women’s Hospital, Harvard Medical School, Boston, USA was cultured as described previously (Fichorova et al., 1997). TZM-bl cell line modified to express CD4 and co-receptors CCR5 and CXCR4, was obtained from the cell repository at National Centre for Cell Science, Pune, India and cultured as reported earlier (Mirani et al., 2019). For HIV-infection assays, activated PBMCs were generated as described previously (Montefiori, 2014).

### Preparation of Viral Stocks

Laboratory-adapted R5-tropic HIV-1 SF162 (subtype B) was obtained from the NIH AIDS Reagent Program, Division of AIDS, NIAID, NIH, from Dr. Jay Levy (Cheng-Mayer and Levy, 1988) and propagated in activated PBMCs as per their protocol (Montefiori, 2014).

### Flow Cytometry

Vaginal epithelial cells were washed twice with PBS and re-suspended in stain buffer (0.2% FBS in PBS). Vk2/E6E7 and TZM-bl cell lines, seeded in T-25 flasks, were detached with 0.25% Trypsin-EDTA (TE), washed with DMEM/F12 containing 10% FBS, before re-suspending in stain buffer. Whole blood subset/cell line staining was carried out as described previously (Prabhu et al., 2019) with fluorochrome-conjugated antibodies including anti-CD4 (clone: RPA-T4), anti-CCR5 (clone: 2D7), anti-CXCR4 (clone: 12G5) and anti-CD206 (clone: 15-2) (BD Biosciences or Biolegend (US)). Stained cells were acquired on BD Accuri C6 flow cytometer and data was analysed on FlowJo 10.6 (BD Biosciences, USA).

### Reverse Transcription PCR 

Cultured Vk2/E6E7 cells were detached using 0.25% T.E. and washed with DMEM/F12 containing 10% FBS before suspension in Ker-SFM. Total cellular RNA was isolated using spin column-based RNA extraction kit (Thermofisher Scientific), followed by cDNA synthesis of ~1µg DNase-treated RNA with SuperScript ® III First-Strand Synthesis System for RT-PCR (Invitrogen) using oligo (dt)s as per manufacturer’s protocol. A nested PCR approach was used to amplify a 201 bp region within the carbohydrate recognizing domain (CRD 4 – 7) of human mannose receptor (Linehan et al., 2001; Fanibunda et al., 2011). Concurrent expression of β-actin gene was evaluated as a housekeeping control (**Supplementary Tables 1–3**). Gene products were resolved on 2% agarose gel and visualized by ethidium bromide staining.

### HIV Binding and Internalization Assays

To examine cell-associated virus, Vk2/E6E7 cells were seeded at a density of 5 x 10^5^ per well (12 well plate) and confluent monolayers were incubated with increasing concentrations (1.25ng – 20ng) of SF162 virus at 37°C overnight. Monolayers were subsequently washed with PBS, detached with 0.25% TE, lysed in protein extraction buffer M-PER (Thermo Scientific™) and assayed for p24 by ELISA (XpressBio). To establish if virus is surface-bound or internalized, monolayers were exposed to virus (1.5ng) at 4°C or 37°C for 1 hour, detached with 0.25% TE and assayed for p24 content by ELISA.

### Detection of Viral RNA

Vk2/E6E7 cells were seeded at a density of 5 x 10^5^/well and confluent monolayers, treated with 1ng SF162, were processed at different time points (15min, 45 min, 90 min, 3 hours) to detect viral RNA. Briefly, virus-exposed cells were washed with 1X PBS, detached with 0.25% TE and total RNA was isolated with Trizol (Invitrogen) based on manufacturer’s protocol. This was followed by cDNA synthesis with SuperScript ® III First-Strand Synthesis System for RT-PCR (Invitrogen) to identify putative viral genomic RNA using HIV LTR-specific primer MSR5. 2 µL of cDNA was used to amplify the C2V3 region of Env gp120 of viral RNA in a nested PCR approach (**Supplementary Table 4**) with primers from the HMA HIV-1 Env subtyping kit obtained through the NIH AIDS Reagents program, Division of AIDS, NIAID, NIH from Dr. James Mullins (Upchurch et al., 2000). Concurrent evaluation of β-actin expression (**Supplementary Table 3**) served as housekeeping control.

### Detection of HIV Reverse Transcription 

Vk2/E6E7 cells were seeded at a density of 1 x 10^5^ cells/well (24 well plate) and confluent monolayers were exposed to DNase (Ambion, Invitrogen) treated viral stock at a concentration of 2.5 ng SF162/well for 2 h at 37°C. Monolayer was extensively washed with 1X PBS and cultured in fresh media. At each time point (24 hours and day-4), cells were detached with 0.25% TE, neutralized with DMEM/F12 containing 10% FBS. Total DNA was isolated using Qiagen DNA extraction kit and viral DNA was detected by amplifying a 232bp sequence from the *gag* gene (Siddappa et al., 2004) and 564bp of Env C2V3 region using a nested PCR approach (**Supplementary Tables 4** and **5**). PHA-p stimulated PBMCs served as positive control and β-actin as housekeeping gene in all PCR assays.

### Productive Infection Assay

To examine productive infection, 1 x 10^5^ Vk2/E6E7 cells/well were seeded and confluent monolayers were treated with 2.5 ng SF162/well for 2 h at 37°C. Thereafter, exposed monolayers were extensively washed with 1X PBS and cultured in fresh media. Day-4 supernatants were assayed for *de novo* viral production by p24 ELISA. PHA-p stimulated PBMCs treated identically served as positive controls for productive infection with HIV.

### Virus Half-Life Assay 

Vk2/E6E7 cells were seeded at a density of 5 x 10^4^ cells/well (96 well plate) and confluent monolayers were treated with non-saturating amounts of HIV-1 SF162 (2.5 ng / well) for 2 hours at 37°C. Cells were washed extensively to remove surface-bound virus, detached with 0.25% TE and cell-associated virus was determined by p24 ELISA over a 48h period as described previously (Gray et al., 2014). Briefly, at each time-point monolayers were detached with 0.25% TE, lysed in protein extraction buffer and assayed for p24 content by ELISA.

### Trans-Infection Assays

Vk2/E6E7 cells were seeded 5 x 10^4^ cells/ well and confluent monolayers were treated with 2.5 ng SF162/well for 2 hours at 37°C. Monolayers were given one wash with 0.05% TE to remove surface-bound virus, extensively washed with 1X PBS and cultured in fresh media. At each time point (0, 3, 6, 24, 48 hours), 2 x 10^5^ PHA-p stimulated PBMCs were added per well and cultured overnight in complete media. Co-cultured PBMCs were recovered and day -4 supernatants were assayed for *de novo* viral production using p24 ELISA. To examine contribution of cell-associated virus to transmission, Vk2 monolayers were exposed to 1.5 ng SF162 at 4°C or 37°C for an hour. Next, both wells were incubated with sCD4 (25 µg/mL, NIH AIDS Reagent Program) at 4°C for 1 hour. Excess inoculum and sCD4 were intermittently washed off with 1X PBS. This was followed by co-culture with PHA-p stimulated PBMCs overnight. Treated PBMCs were retrieved and day -4 supernatants were assayed for p24. To inhibit lymphocyte adhesion to epithelial cells, stimulated PBMCs were pre-incubated with 2 µg/mL anti-LFA-1 antibody (anti-CD18, Abcam, clone: MEM-148) for 30 mins, prior to co-culture with virus-exposed Vk2 cells.

### Sorting of T Cell Subsets

Freshly isolated PBMCs from healthy donors were stained with fluorochrome conjugated antibodies – anti-CD3 (Clone: SK7), anti-CD4 (Clone: RPA-T4), anti-CCR5 (Clone: NP6G4), anti-CXCR4 (Clone: 12G5) and anti-β7 (Clone: FIB504) for 20 mins at 37°C. Cells were washed and re-suspended in complete medium (RPMI-1640 + 10% FBS + 1X PenStrep) with 5 units/mL IL-2. Cells were sorted on the BD FACS ARIA FUSION into flow tubes coated with FBS applying a gating strategy for T cell subsets shown in **Supplementary Figures 1A, B**. Detailed workflow for trans-infection is shown in **Supplementary Figure 2**.

### Statistical Analysis

Statistical comparisons were made using two-tailed Student’s t-test or Mann-Whitney non-parametric test with a P value less than 0.05 being considered significant.

## Results

### Expression of HIV-Binding Receptors on Primary Cells and Vk2/E6E7 Cell Line

**Figure 1:**
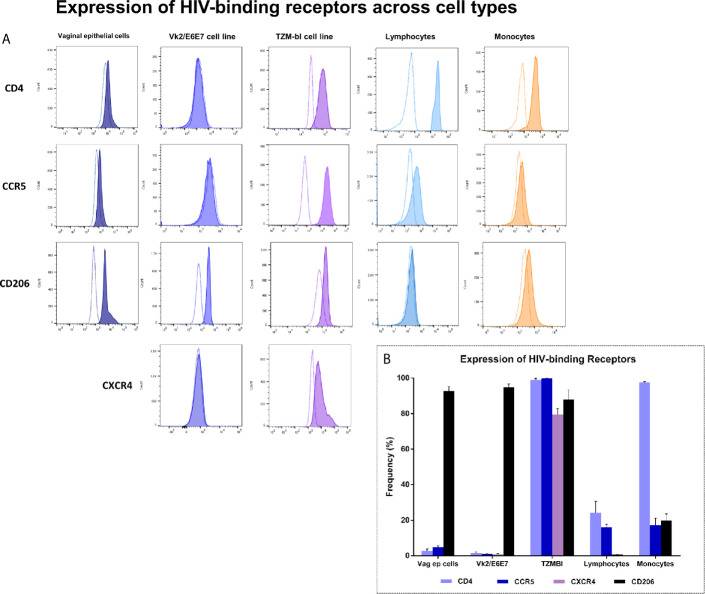
Expression of HIV-binding receptors across cell types – Primary vaginal epithelial cells, lymphocytes and monocytes, as also Vk2/E6E7 and TZM-bl (positive control for CD4, CCR5 and CXCR4 expression) cell lines were examined for their expression of HIV-specific receptors CD4, CCR5, CXCR4 and human mannose receptor (CD206) by flow cytometry. **(A)** Representative histograms showing the expression of CD4, CCR5, CD206 and CXCR4 across various cell types. Plots were generated using FlowJo version 10.6; stained samples (filled histograms) were superimposed on unstained controls (dotted clear histogram). **(B)** Data is represented as frequency of cells expressing the receptor (percentage positivity) in a minimum of three independent experiments for cell lines. Data for vaginal epithelial cells is obtained from vaginal swabs (n = 7) and for lymphocytes and monocytes is obtained by whole blood staining (n = 6). Bars indicate mean ± standard error for mean (SEM).

To establish concordance between primary vaginal epithelial cells and Vk2/E6E7 cell line in terms of HIV-binding receptor expression, vaginal swab samples as well as blood of seronegative volunteers (SN, n = 4) and HIV-1 infected individuals (PA, n = 4; ART, n = 5) was immunophenotyped. Lymphocyte and monocyte subsets were examined by whole blood staining. Clinical characteristics of the participants have been summarized in **Supplementary Table 6**. Vaginal epithelial cells were gated based on scatter and examined for their expression of HIV-binding receptors CD4, CCR5 and CD206 compared to unstained controls (**Supplementary Figure 3**). T-lymphocytes and monocytes were identified based on forward and side scatter properties and expression of CD3 and CD14/CD16 respectively as described previously (Prabhu et al., 2019). **Figure 1A** depicts representative histograms of HIV-specific receptor expression across these cell types. We observed, as expected, that primary vaginal epithelial cells as well as Vk2/E6E7 cell line demonstrated negligible levels of HIV-binding receptor, CD4 and co-receptors, CCR5 and CXCR4 compared to ex-vivo stained T-lymphocytes and monocytes or TZM-bL cells respectively (**Figure 1B** and **Supplementary Table 7**).

Interestingly, CD206, a marker of alternate activation (M2 phenotype) observed on blood monocytes as reported earlier by our group (Prabhu et al., 2019) was observed to be highly expressed both on primary vaginal epithelial cells and epithelial cell lines (Vk2/E6E7 and TZM-bL) (**Figure 1B** and **Supplementary Figure 4**). These results highlighted the suitability of using the VK2/E6E7 cell line to study CD4-independent interaction of HIV with the vaginal epithelium.

### Sequestration of HIV-1 by Vaginal Epithelial Cells 

**Figure 2:**
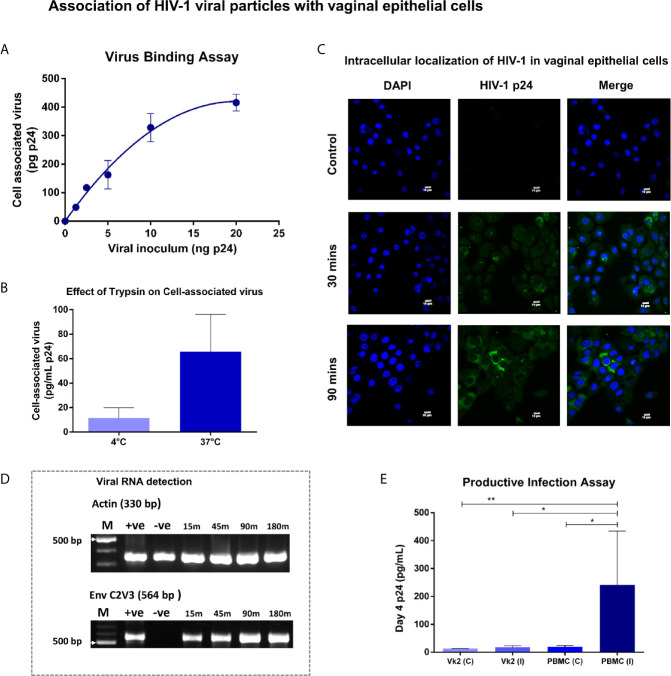
Association of HIV-1 viral particles with vaginal epithelial cells – **(A)** Virus binding assay - Bound virus was plotted as a function of viral inoculum (mean ± standard error) after subtracting background. Dose-dependent increase in cell-associated virus approached saturation above 10ng. **(B)** Bar graph demonstrating cell-associated virus at 4°C and 37°C showed substantial decline (P = 0.06) in cell-associated virus at 4°C compared to physiological temperatures (37°C). **(C)** Vk2/E6E7 cells exposed to SF162 (2ng p24) for 30 and 90 minutes at 37°C respectively, were washed extensively (1X PBS), fixed and stained with anti-HIV-1 p24 antibody KC57 (FITC) and counterstained with DAPI. Immunofluorescence images were taken on an Olympus Fluorview FV3000 microscope with 405 nm (DAPI) and 488 nm (FITC) lasers for excitation and a 60x/1.42 oil immersion objective for acquisition. Images were processed using Fluoview software. Intracellular localization of HIV-1 (FITC) was observed in vaginal epithelial cells, 30 minutes’ (middle panel) post-viral incubation that became more pronounced on increasing the duration of viral exposure to 90 minutes (bottom panel). Unexposed Vk2/E6E7 cells served as negative control (top panel). **(D)** Viral RNA detection - Time-point assay using nested PCR up to 3 h post- viral exposure for Env C2V3 and β-actin in Vk2/E6E7 cell line. **(E)** Productive infection assay - Day-4 supernatants for Vk2/E6E7 cell line exposed to SF162 [Vk2 (I)] had insignificant p24 levels compared to uninfected control [Vk2 (C)]. Activated PBMCs [PBMC (I)] had highly significant p24 levels compared to uninfected control [PBMC (C)] and VK2(I). Results are representative of at least three independent experiments. Statistical significance was estimated by Students t-test for paired samples or Mann-Whitney non-parametric test for comparison between groups; *P < 0.05, **P < 0.01.

The interaction of HIV-1 with vaginal epithelial cells was examined by titrating Vk2/E6E7 monolayers with a range of viral concentrations (0 – 20 ng p24 of SF162) at 37°C. We observed a dose-dependent increase in cell-associated virus that approached saturation above ~10 ng p24 (**Figure 2A**). In order to determine whether detectable p24 in total cell lysates was attributable to internalized virus, Vk2/E6E7 cells were exposed to non-saturating amounts of viral inoculum at 4°C and 37°C respectively. At low temperatures, internalization of virions is arrested and trypsin can cleave off surface-bound virus (Herrera et al., 2016). Indeed, in our system we observed a measurable decline in cell-associated virus at 4°C compared to physiological temperatures (37°C) (**Figure 2B**). We also observed viral uptake by immunofluorescence staining of SF162-exposed Vk2/E6E7 cells using anti-HIV-1 p24 antibody KC57 (FITC) (**Figure 2C**). This suggested that viral uptake was a metabolically active process in vaginal epithelial cells which in turn sequester virions in intracellular compartments.

Next, to evaluate if sequestered virus contained viral RNA and was thus potentially infectious, we performed reverse transcription PCR to generate cDNA, followed by amplification of HIV-1 *env* gene using a nested PCR assay. Indeed, the C2V3 region of viral envelope, significant for its role in co-receptor tropism (X4/R5), could be successfully amplified using this approach. This semi-quantitative assay showed increasing intensity of Env C2V3 band observed to reach saturation ~2 hours' post-exposure to viral inoculum (**Figure 2D**). Interestingly, a band was visible at the earliest time-point as well indicating that viral uptake by vaginal epithelial cells occurred rapidly. β-actin gene served as the housekeeping control in all PCR assays (**Figure 2D**). Thus, we were able to demonstrate the presence of both viral protein and RNA in vaginal epithelial cells post-exposure to HIV-1 SF162.

### Vaginal Epithelial Cells Are Not Productively Infected by HIV

To establish if viral uptake led to a productive infectious cycle, Vk2/E6E7 cells were exposed to SF162 for 2 hours at 37°C, extensively washed and cultured in fresh medium for 4 days, after which, supernatants were analysed for p24 levels. We detected negligible amounts of p24 in the supernatants of vaginal epithelial cells that was insignificant compared to uninfected controls (**Figure 2E**). Conversely, PHA-p stimulated PBMCs concurrently exposed to SF162, which served as the positive control, showed significantly higher p24 (P<0.05) levels compared to uninfected controls and exposed Vk2/E6E7 cells (P<0.05) (**Figure 2E**). 

Also, Vk2/E6E7 cells exposed to DNAse-treated viral stocks (2.5 ng SF162) did not exhibit amplification of the *gag* gene or *env* gene clearly visible in similarly treated PHA-p stimulated PBMCs (**Supplementary Figure 5**). This further suggested that sequestration of virus was probably not followed by reverse transcription and subsequent steps in the viral life cycle within vaginal epithelial cells.

### Vaginal Epithelial Cells as Transient Reservoirs for HIV

**Figure 3:**
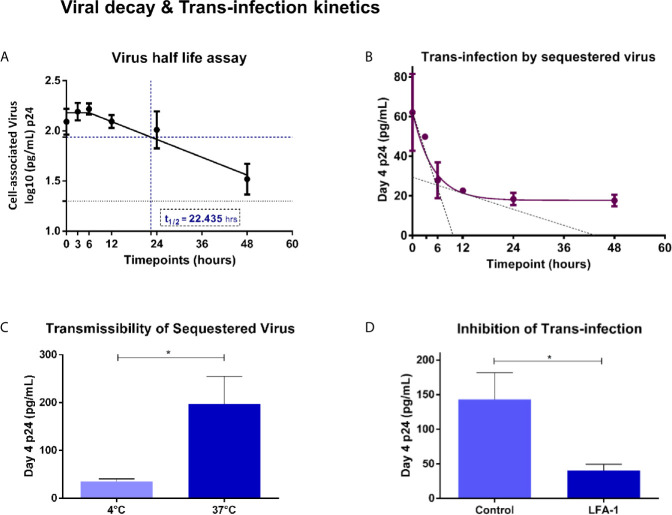
Viral decay and Trans-infection kinetics – **(A)** Virus half-life assay – Exponential monophasic decay of sequestered virions with a half-life of 22.435 hours (interpolated from graph – blue dash) in Vk2/E6E7 cells over a 48 h time course. Residual virus was detectable (black dotted line indicates limit of detection for assay [20pg/mL]) at the final time point (48h). **(B)** Trans-infection by sequestered virus – Bi-phasic decay of p24 levels in day-4 supernatants of co-cultivated PBMCs (fast half-life = 3.33 h; slow half-life = 11.82 h interpolated from the graph, indicated by black dotted line). **(C)** Transmissibility of sequestered virus – Bar graph demonstrating trans-infection of cell-associated virus at 4°C and 37°C. **(D)** Inhibition of trans-infection - Bar graph demonstrating inhibition of trans-infection by blocking LFA-1 receptor on PBMCs. Results are representative of at least three independent experiments. Statistical comparisons were made using paired t test, *P < 0.05.

In order to understand the implications, if any, of viral uptake in the possible transmission, through trans-infection, of HIV-1 to target cells we first sought to determine the kinetics of retention of sequestered virions by vaginal epithelial cells over a 48-hour time period. At each time-point, post-incubation with viral inoculum, Vk2/E6E7 cells were assayed for intracellular virus. We observed monophasic decay after 6 hours with a half-life of 22.435 hours (**Figure 3A**). To ascertain the infectivity of sequestered virus, SF162 exposed-Vk2/E6E7 cells were co-cultured with activated PBMCs from a healthy donor at different time points. Day-4 supernatants of co-cultured PBMCs were harvested and assayed for p24 levels. We observed that ability of vaginal epithelial cells to harbour infectious virus deteriorated with time, as evidenced by the decline in p24 values of supernatants from co-cultivated PBMCs (**Figure 3B**). Trans-infection kinetics demonstrated a biphasic decay of functional virus with an initial rapid half-life of 3.33 hours followed by a slower half-life of ~11.82 hours (**Figure 3B**). Interestingly, when we super-imposed graphs depicting trans-infection kinetics with viral decay in epithelial cells, we observed that peak levels of sequestered virus coincide with maximum trans-infection as evidenced by higher p24 levels in supernatants of co-cultivated PBMCs (**Figures 3A, B**). However, although residual virus was detectable in vaginal epithelial cells even at 48 hours’ post-exposure, trans-infection efficiency steadily deteriorated revealing a narrow but distinct window for transmissible virus to infect susceptible cells (**Figures 3A, B**).

### Trans-Infection Mediated by Sequestered Virus Is Inhibited by Blocking LFA-1 

In order to assess the contribution of surface bound v/s internally sequestered virus in transmission to co-cultured PBMC, we exposed Vk2/E6E7 cells to SF162 virus at 4°C and 37°C and treated them with sCD4 that would bind (gp120) and inhibit transmission of surface-bound virus but not that of virus sequestered intracellularly as has been previously reported (Yasen et al., 2018). Activated PBMCs co-cultured with Vk2/E6E7 cells exposed to virus at 37°C, a condition permissive to intracellular uptake (also see **Figure 2B**), displayed significantly higher levels of p24 in day-4 supernatant than those exposed at 4°C (**Figure 3C**), thereby demonstrating trans-infection by sequestered virus in Vk2/E6E7 cells.

Further, a lymphocyte receptor function-associated antigen 1 (LFA-1) dependent mechanism has been implicated in cell-to-cell spread of HIV between primary epithelial cells (explants) and PBMCs as well as CD4+T cells (Yasen et al., 2017). To ascertain the role of LFA-1 in our trans-infection model, we pre-treated activated PBMCs with anti-LFA-1 antibody prior to co-culture with HIV exposed Vk2/E6E7 cells. Blocking LFA-1 significantly reduced viral output of treated PBMCs following co-culture compared to untreated (control) cells as evidenced by a decline in p24 values of day-4 supernatants (**Figure 3D**).

### Trans-Infection of Sorted T Cell Subsets by Vaginal Epithelial Cells

**Figure 4:**
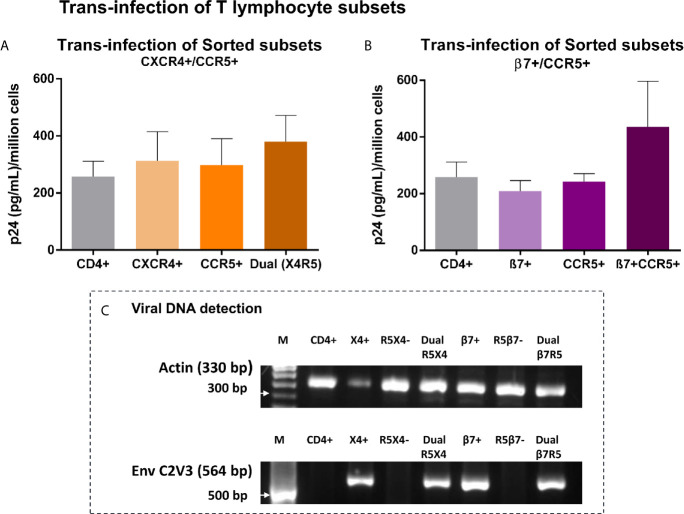
Trans-infection of T lymphocyte subsets – **(A, B)** Bar graphs showing p24 levels of supernatants recovered from T lymphocyte subsets, sorted based on expression of HIV-1 co-receptors CXCR4 and CCR5 **(A)**, AND integrin β7 and HIV-1 co-receptor CCR5 **(B)** following co-culture with virus-exposed Vk2/E6E7 cells. Dual-positive cells produced considerably higher p24 than cells singly positive for either receptor, highlighting the significance of this target cell-type in mucosal transmission. Results are representative of three independent experiments. Statistical comparisons were made using paired t test. **(C)** Detection of viral DNA in T lymphocyte subsets recovered post co-culture with SF162-exposed Vk2/E6E7 cell line by amplifying HIV-1 Env C2V3 region accompanied by β-actin controls.

To putatively recapitulate the early events in vaginal transmission of HIV we evaluated the transmission of sequestered virus to specific populations of CD4+T cells known to be permissive to infection. Vk2/E6E7 cells previously exposed to SF162, were co-cultured with FACS sorted CD4+ T lymphocytes or subsets thereof based on differential expression of HIV-binding co-receptors CCR5 and CXCR4 and gut-homing receptor - integrin α4β7. As shown in the first panel, we observed that CD4+ T lymphocytes enriched for both CXCR4 and CCR5 had marginally higher (not significant) p24 levels in recovered supernatants than CD4+ T cells as a whole (**Figure 4A**). Interestingly, for the second panel, CD4+ T cells dual-positive for co-receptor CCR5 and β7 were conducive to trans-infection as evidenced by elevated, though not statistically significant, p24 levels in culture, highlighting a role for β7 in facilitating viral attachment to these cells (**Figure 4B**).

Further, we were able to confirm actual infection of subsets through detection of viral DNA in co-cultivated subsets singly positive for CXCR4, integrin β7, and dually positive for both CCR5/CXCR4 and β7/CCR5 by amplifying the C2V3 region of the *env* gene with β-actin serving as housekeeping control (**Figure 4C**). Therefore, we could demonstrate trans-infection of CD4+ T lymphocyte subsets solely mediated by vaginal epithelial cells in the absence of accessory cells such as monocytes and dendritic cells otherwise present in a stimulated PBMC population.

## Discussion

In this study, we examined interaction of HIV-1 with vaginal epithelium, widely regarded as the primary site for viral transmission, using Vk2/E6E7, an established cell line extensively used to model STIs/RTIs (Pandit et al., 2019; Young et al., 2019; Brannon et al., 2020). Vk2/E6E7 exhibited an HIV-binding receptor phenotype (CD4-, CCR5-) similar to primary vaginal epithelial cells obtained from swab samples, as observed by flow cytometry. Notably, both cell line and primary cells expressed human mannose receptor (hMR, CD206), an alternate HIV-1 receptor (Nguyen and Hildreth, 2003; Lai et al., 2009; Sukegawa et al., 2018) corroborating our previous work demonstrating presence of hMR mRNA and protein expression in these cell types (Fanibunda et al., 2011).

In concordance with earlier reports on vulvo-vaginal cell lines (Kohli et al., 2014), we observed dose-dependent increase in viral uptake by vaginal epithelial cells on exposure to viral inoculum that was temperature-dependent and resistant to trypsin cleavage. Together with detection of viral capsid p24 in total cell lysates and viral RNA we demonstrate presence of intact virions in vaginal epithelial cells. Semi-quantitative nested PCR revealed viral RNA at the earliest time point (~15 min) which reached saturation ~2 hours’ post-exposure. In a previous *in vivo* study of vaginal transmission, SIV penetration from the lumen to vaginal mucosa of RMs occurred within 60 mins of inoculation resulting in productive infection (Hu et al., 2000). Also, Hope and colleagues demonstrated a positive correlation between viral concentration of the inoculum and depth of vaginal tissue penetration (Carias et al., 2013). We similarly observed a dose dependent increase in cell-associated virus that achieved saturation at 10 ng p24 (absolute) of viral inoculum with up to ~5.35% virion retention by the cells corresponding to ~5 million copies. Viral sequestration, however, was not followed by successful reverse transcription and *de novo* production of HIV in vaginal epithelial cells unlike in activated PBMCs as shown before for non-vaginal epithelial cells (Wu et al., 2003; Kohli et al., 2014).

To better understand the kinetics of viral retention we determined the half-life of sequestered virus in vaginal epithelial cells and observed an exponential decline after ~6 hours with residual virus detectable up to 48 hours’ post-exposure. Interestingly, SIV-exposed RMs also exhibit a substantial reduction in detectable virus in the female reproductive tract (FRT) 12 h after inoculation, suggesting the presence of mechanisms *in vivo* to eliminate virus (Carias et al., 2013).

Paracellular penetration of HIV in the vaginal mucosa is an inefficient process *in vitro* and remains to be demonstrated *in vivo* (Tugizov, 2016; Gonzalez et al., 2019). Exposure to HIV-1 is known to induce gp120-mediated disruption of the epithelial barrier in oral, intestinal and genital mucosae (Nazli et al., 2010; Lien et al., 2019). In support of these findings, we observed significant reduction in trans-epithelial electrical resistance (TER) of the vaginal epithelial monolayer on exposure to SF162 over time (**Supplementary Figure 6**), signalling loss of barrier integrity. As intraepithelial CD4+ T lymphocytes occur at low abundance, and target cells expressing CD4 and CCR5 are more likely to populate the basal epithelium and underlying *lamina propria* (Poonia et al., 2006), epithelial cells as ‘transient’ reservoirs could enhance the probability of acquiring infection. Interestingly, previous studies using polarized epithelial cells demonstrated presence of sequestered virions even 6-9 days’ post-exposure (Yasen et al., 2017). The observed retention window for captured virus in our system, albeit lower than primary cells, was sufficient for onward transmission, as evidenced by infection of activated PBMCs co-cultivated with SF162-exposed vaginal epithelial cells. As expected, the ability of epithelial cells to sustain a transmissible viral reservoir also deteriorated with time, reflected in a reduction of trans-infection efficiency. Previously, Yasen et al demonstrated that cell-to-cell HIV spread is mediated by the specific interaction of intercellular adhesion molecule-1 (ICAM-1) on epithelial cells with lymphocyte receptor function-associated antigen-1 (LFA-1) on activated PBMCs using cervical and foreskin epithelial cells (Yasen et al., 2017). A similar role for LFA-1 was observed in our model system whereby blocking the receptor on PHA-p stimulated PBMCs with anti-LFA-1 antibody significantly diminished transmission of sequestered virus from vaginal epithelial cells.

We further investigated the ability of vaginal epithelial cells to infect, *in trans*, not just activated PBMCs but also susceptible target CD4+ T lymphocyte subsets in isolation. Notably, we demonstrated transmission of sequestered virus to sorted T cell subsets that were not extraneously activated and therefore reminiscent of an *in vivo* transmission scenario. Vaginal CD4+ T cells have been shown to express CCR5 (also co-express CXCR4) abundantly, most exhibiting a ‘central memory’ phenotype (~95%) and these are preferentially depleted in SIV infection as observed in macaque models (Veazey et al., 2003; Poonia et al., 2006). In our study, we observed that vaginal epithelial cells did facilitate infection of sorted CD4+ T cell subsets enriched for co-receptors CCR5/CXCR4. Integrin α4β7 is a gut homing receptor implicated in massive depletion of CD4+ T cells in intestinal lymphoid tissue during the acute phase of HIV infection (Brenchley et al., 2004; Li et al., 2005; Mattapallil et al., 2005; Arthos et al., 2008). A study by Byrareddy et al demonstrated the protective effects of anti-a4b7 monoclonal antibody following repeated low-dose intravaginal challenges with SIV in rhesus macaques (Byrareddy et al., 2014). α4β7+ CD4+ T lymphocyte subset thus plays a crucial role in mucosal transmission (Cicala et al., 2009; Sivro et al., 2018) and we report for the first time that CD4+ T cells expressing both β7 and CCR5 were conducive to trans-infection following co-culture with HIV-1 exposed vaginal epithelial cells.

In this study, we attempt to recapitulate the early events in vaginal transmission of HIV using an established vaginal epithelial cell line. One of the key limitations is the absence of polarized structure in our study model (monolayer of cells), otherwise inherent in epithelial tissues and future studies would involve three-dimensional modelling of vaginal epithelium to better understand host-microbe interactions. We also acknowledge the importance of examining the influence of vaginal microbiome on HIV acquisition. Our model system provides a cost-effective and possibly adequate alternative to the use of explants and non-human primate models in preliminary studies to evaluate therapeutic candidates for Prep and microbicide development. It also explores a novel role for the vaginal epithelium, historically thought to be a barrier to pathogens, as a dynamic tissue system capable of facilitating HIV transmission.

## Data Availability Statement

The original contributions presented in the study are included in the article/**Supplementary Material**. Further inquiries can be directed to the corresponding author.

## Ethics Statement

The studies involving human participants were reviewed and approved by NIRRH Institutional Clinical Ethics Committee (project No. 160/2009 & No. 225/2012). The patients/participants provided their written informed consent to participate in this study.

## Author Contributions

VPat, VPra and AB were involved in study design, data analysis and writing the original manuscript. VPad, VPra and SV enrolled participants and VPad collected vaginal swabs. VPra performed experiments. SS aided in analysis of HIV-binding receptor flow cytometry data. VN and PP enabled participant recruitment and collection of clinical history. VPat and VPra reviewed and edited the final manuscript. All authors contributed to the article and approved the submitted version.

## Funding

This study was funded by Department of Biotechnology (DBT), India with grant no. (BT/PR6202/GBP/27/383/2012) awarded to the corresponding author VPat and intramural funding provided by ICMR, India. VPra is a recipient of Junior Research Fellowship from University Grants Commission (UGC), Govt. of India. The funding agencies had no role in study design, sample collection, data analysis, or preparation of manuscript.

## Conflict of Interest

The authors declare that the research was conducted in the absence of any commercial or financial relationships that could be construed as a potential conflict of interest.

## Abbreviations

HIV, human immunodeficiency virus; PLHIV, people living with HIV; ART, antiretroviral therapy; PreP, pre-exposure prophylaxis; FRT, female reproductive tract; hMR, human mannose receptor; RM, rhesus macaque; SIV, simian immunodeficiency virus; PA, Pre-ART; PBMC, peripheral blood mononuclear cells; PHA, phytohemagglutinin; LFA-1, lymphocyte receptor function-associated antigen-1; TE, Trypsin-EDTA; ELISA, Enzyme-linked immunosorbent assay; FACS, Fluorescence-activated cell sorting.
