# Supplementary material for: Vaginal Epithelium Transiently Harbours HIV-1 Facilitating Transmission
Source: Front Cell Infect Microbiol. 2021 Mar 17;11:634647. doi: 10.3389/fcimb.2021.634647 (PMC8011497; doi:10.3389/fcimb.2021.634647)
Supplement: Supplementary file 1 [file DataSheet_1.pdf]

Supplementary Data
(Tables)

Supplementary table 1 : List of primers

Gene	Sense primer	Anti-sense primer	Product
**β Actin**	5' – GCTCGTCGTCGACAACGGCTC – 3'	5' – CAAACATGATCTGGGTCATCTTCTC– 3'	**330 bp**
**hMR**	1st round<br>5'-GAGGATCCAGGACCCAAATGTCCAGAAAACTGG-3'<br><br>2nd round<br>5' – TACACAAACTGGGGGAAAGG – 3'	1st round<br>5'- GAGGATCCACTTACCTGTGAAAGGAATCCACGCAGTC TGTTC – 3'<br><br>2nd round<br>5' – TGTTTGAATCGTTGCTGGAG – 3'	**201 bp**
**Gag**	1st round<br>5' – GATGGTGCTTCAAGCTAGTRCCAGTTGA – 3'<br><br>2nd round<br>5' – CTCTCGACGCAGGACTCGGCTTGCTGA – 3'	1st round<br>5' – CTCTATYTTRTCTAARGCTTCYTTGGTGTC -3'<br><br>2nd round<br>5' – TTCYAGCTCCCTGCTTGCCCATACTA – 3'	**232 bp**
**Env C2V3**	1st round (ED5)<br>5' - ATGGGATCAAAGCCTAAAGCCATGTG – 3'<br><br>2nd round (ED31)<br>5' –CCTCAGCCATTACACAGGCCTGTCCAAAG –3'	MSR5 (cDNA synthesis)<br>5' – GCACTCAAGGCAAGCTTTATTGAGGCTT -3'<br><br>1st round (ED12)<br>5' - AGTGCTTCCTGCTGCTCCCAAGAACCCAAG – 3'<br><br>2nd round (ED33)<br>5' – TTACAGTAGAAAAATTCCCCTC – 3'	**564 bp**

Supplementary table 2: hMR (201) PCR

Reagents	1st Round
Roche Buffer 2 (27.5 mM $MgCl_2$)	1X
dNTPs	1.8mM
Sense primer	0.4µM
Anti-sense primer	0.4µM
Roche enzyme*	5U
cDNA	3µL
	50µL react^n

*Roche Expand Long Template PCR system

Initial denaturation	94° C	2 m
10 cycles	94° C	30 s
	62° C	30 s
	68° C	2 m
25 cycles	94° C	30 s
	60° C	30 s
	68° C	2 m
Final extension	72° C	10 m

Reagents	2nd Round
Taq buffer	1X
dNTPs	1 mM
$MgCl_2$	3 mM
Sense primer	0.4µM
Anti-sense primer	0.4µM
Taq pol ^	5U
1st product	10µL
	25µL react^n

^Bangalore Genei (India)

Initial denaturation	94° C	2 m
35 cycles	94° C	15 s
	58° C	30 s
	68° C	2 m
Final extension	68° C	5 m

Supplementary table 3: β Actin PCR

**Reagents**	
Taq buffer	1X
dNTPs	1.25 mM
$MgCl_2$	3 mM
Sense primer	1 µM
Anti-sense primer	1 µM
Taq pol^	5U
Template/cDNA	~100ng DNA / 2µL
	20µL react^{n}

^Bangalore Genei (India)

Cycling parameters		
Initial denaturation	94° C	2 m
35 cycles	94° C	30 s
	56° C	1 m
	68° C	1 m
Final extension	68° C	7 m

Supplementary table 4: Env C2V3 PCR

**Reagents**	1st & 2nd Round
Taq buffer	1X
dNTPs	1 mM
$MgCl_2$	3 mM
Sense primer	0.3 µM
Anti-sense primer	0.3 µM
Taq pol^	5U
Template/cDNA/ 1st product	~100ng DNA / 2µL / 10µL
	25µL react^n

^Bangalore Genei (India)

Cycling parameters		
3 cycles	94° C	1 m
	55° C	45 s
	72° C	1 m 40 s
32 cycles	94° C	15 s
	55° C	45 s
	72° C	1 m
Final extension	72° C	5 m
Hold	4° C	

Supplementary table 5: Gag PCR

**Reagents**	1st Round
Taq buffer * (27.5 mM $MgCl_2$)	1X
dNTPs	1.25 mM
Sense primer	0.5 µM
Anti-sense primer	0.5 µM
Taq pol*	2.5U
Template	~100ng DNA
	20µL react^n

*Geneall (India)

Cycling parameters		
3 cycles	94° C	1 m
	60° C	1 m
	72° C	1 m
25 cycles	94° C	15 s
	60° C	45 s
	72° C	1 m
Final extension	72° C	5 m

**Reagents**	2nd Round
Taq buffer * (27.5 mM $MgCl_2$)	1X
dNTPs	1 mM
Sense primer	0.4 µM
Anti-sense primer	0.4 µM
Taq pol*	2.5U
1st product	10µL
	25µL react^n

*Geneall (India)

Cycling parameters		
3 cycles	94° C	1 m
	65° C	40 s
	72° C	40 s
35 cycles	94° C	1 m
	65° C	40 s
	72° C	40 s
Final extension	72° C	5 m

Supplementary table 6 : Clinical Characteristics of Participants

	Seronegative (n = 4)	Pre-ART (PA) (n = 4)	ART (n = 5)
Median age, years	36 (26 – 38)	29 (24 – 35)	38 (36 – 45)
Median CD4 count, cells/µL	1254	329 (290 -522)	396.5 (184 – 819)
Median viral load, log (copies/mL)	-	4.79 (4.01 – 6.27)	UD‡ (1), 3.41 (2.55 – 4.26)
Median duration of infection˜, years	-	0.5 (0 – 4)	9.67 (2.16 – 11)

Data expressed as median followed by range. Absolute CD4 counts were not available for 5 individuals.
Viral load data was unavailable for 2 individuals on ART.

‡ Undetectable (<34 copies/mL)

˜ Duration of infection was estimated from date of diagnosis

Supplementary table 7: Expression of HIV-binding receptors by flow cytometry

	Vaginal epithelial cells	Vk2/E6E7	TZM-bl	Lymphocytes	Monocytes
CD4	**1.23 %** (0.6 -5.18%)	**1.82 %** (0.25 – 2.64 %)	**99.7 %** (97 – 100%)	**19 %** (9.48 – 38.4%)	**97.7 %** (97.03 – 98.3%)
CCR5	**4.76 %** (3.94 -5.59%)	**0.82 %** (0.5 – 1.74%)	**100 %** (99.7 – 100%)	**14.90 %** (13.23 – 20.28%)	**14.5%** (8.91 – 28.75%)
CD206	**93.2 %** (86.28 – 98.75%)	**95.20 %** (91.6 – 97.9%)	**87.0%** (78.8 – 97.9%)	**0.74 %** (0.54 – 0.91%)	**18.65 %** (11.53 – 29.3%)
CXCR4	--	**1.23 %** (0.24 – 1.27%)	**82.5 %** (72.7 – 83.1%)	--	--

Values represent percentage positivity expressed as median [interquartile range]. CXCR4 expression was only examined for cell lines (Vk2/E6E7 and TZM-bl).
