# Supplementary material for: Vaginal Epithelium Transiently Harbours HIV-1 Facilitating Transmission
Source: Front Cell Infect Microbiol. 2021 Mar 17;11:634647. doi: 10.3389/fcimb.2021.634647 (PMC8011497; doi:10.3389/fcimb.2021.634647)
Supplement: Supplementary file 2 [file DataSheet_2.pdf]

Supplementary Data

(Figures)

Supplementary Fig. 1a : Gating strategy for sorting CXCR4/CCR5 expressing CD4+ T lymphocyte subsets

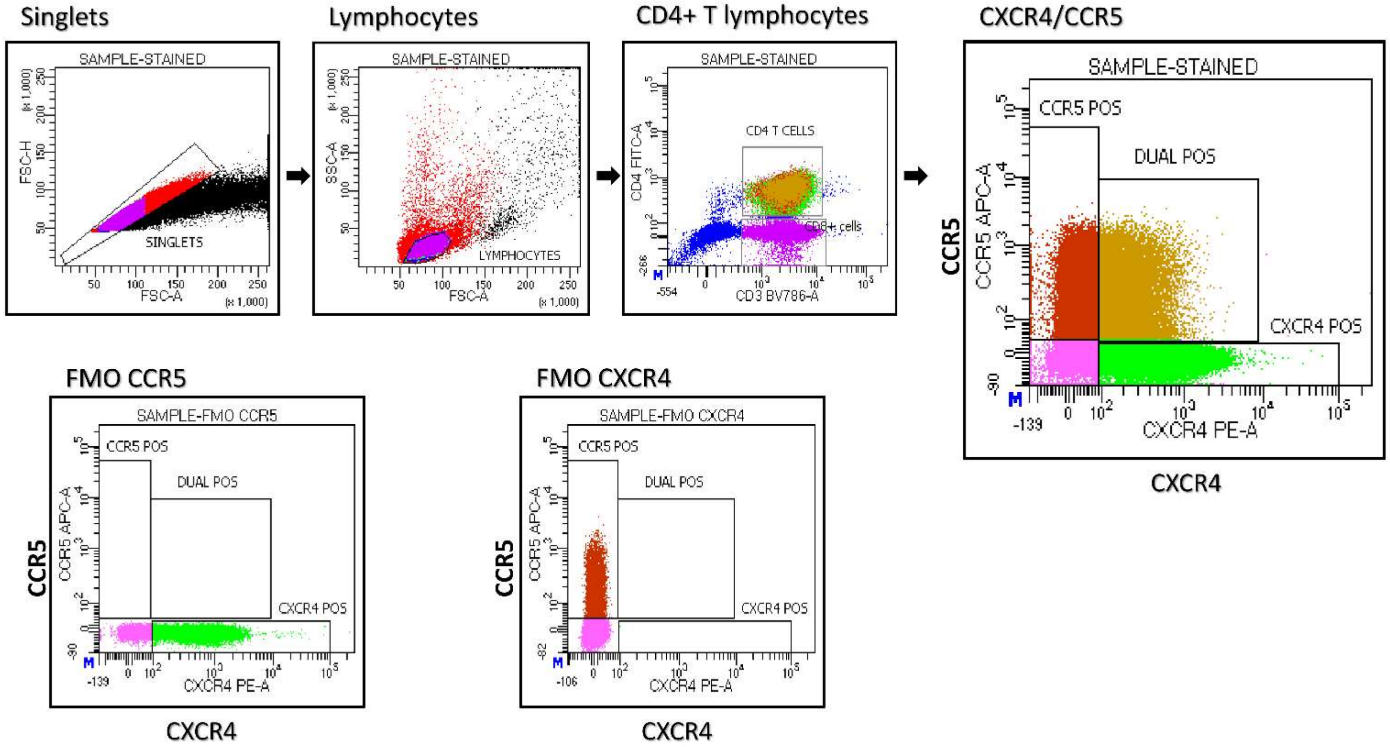

<u>Supplementary Fig. 1a</u> - Representative plots displaying gating strategy for FACS sorting of CD4+ T lymphocyte subsets expressing co-receptors CXCR4 and CCR5 with fluorescence minus one (FMO) controls. Three populations were sorted; CD4+ T cells singly positive for CXCR4, singly positive for CCR5 and dual-positive for both co-receptors.

Supplementary Fig. 1b : Gating strategy for sorting β7/CCR5 expressing CD4+ T lymphocyte subsets

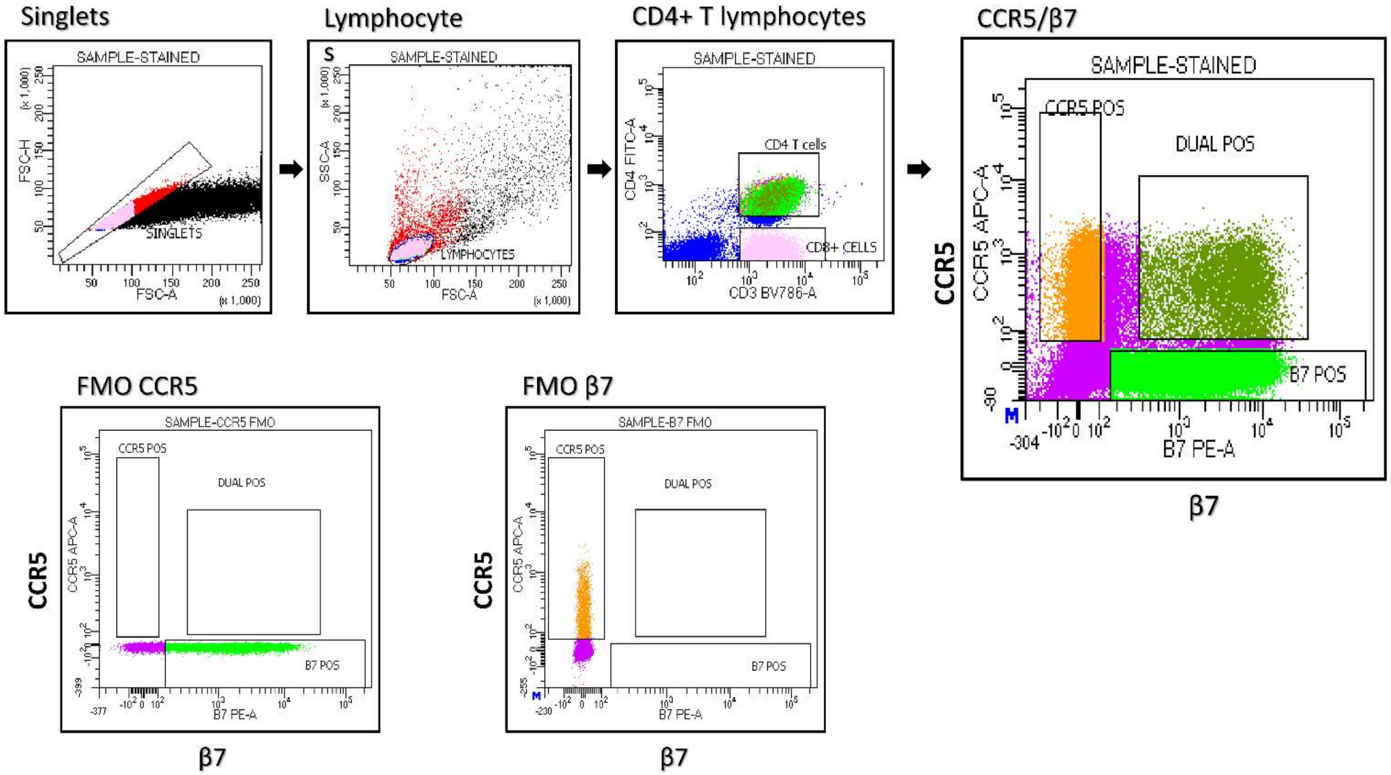

Supplementary Fig. 1b - Representative plots displaying gating strategy for FACS sorting of CD4+ T lymphocyte subsets expressing integrin β7 and co-receptor CCR5 with fluorescence minus one (FMO) controls. Three populations were sorted; CD4+ T cells singly positive for β7, singly positive for CCR5 and dual-positive for both.

Supplementary Fig.2: Workflow for trans-infection of T lymphocyte subsets

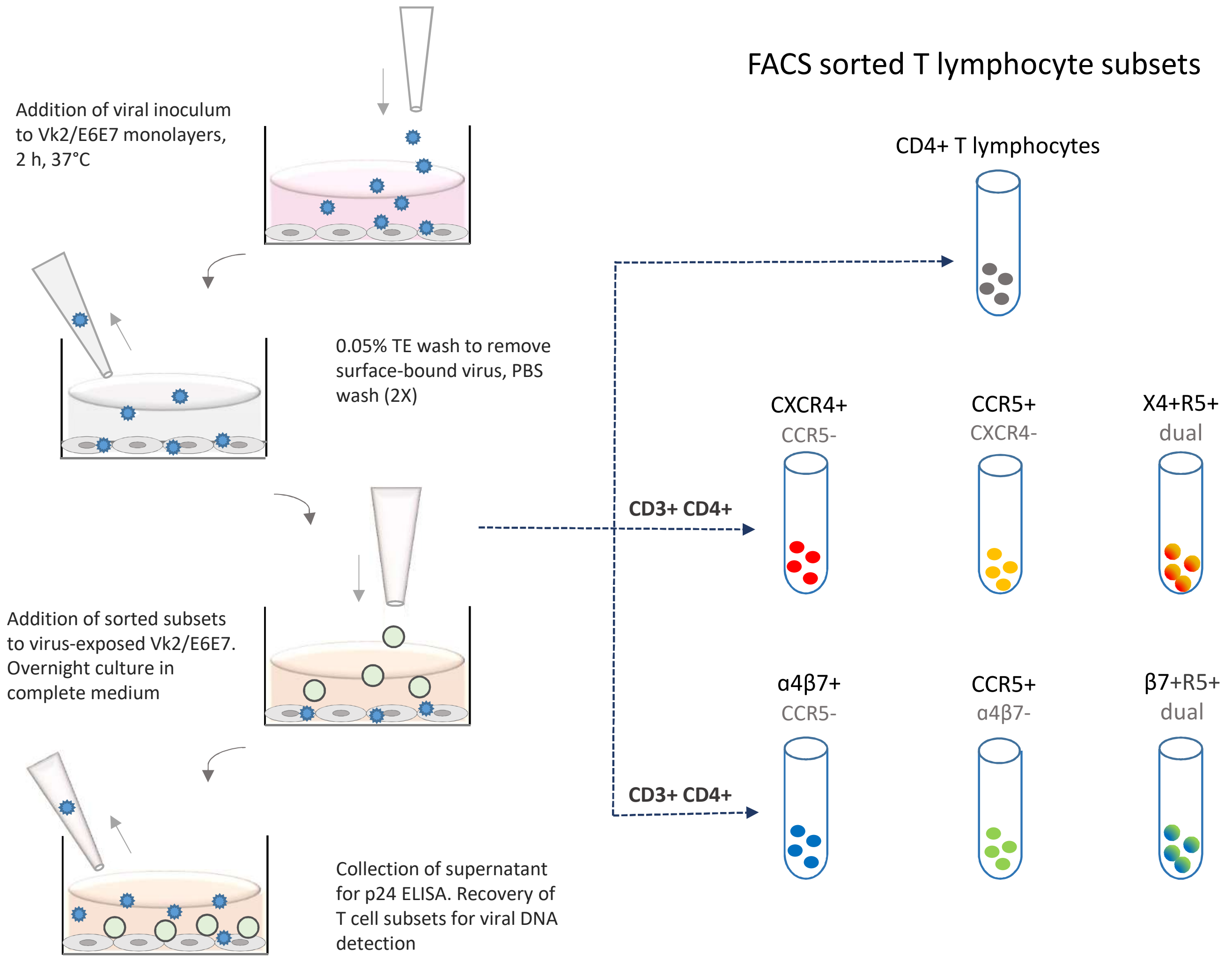

Supplementary Fig.3: Gating strategy for vaginal epithelial cells

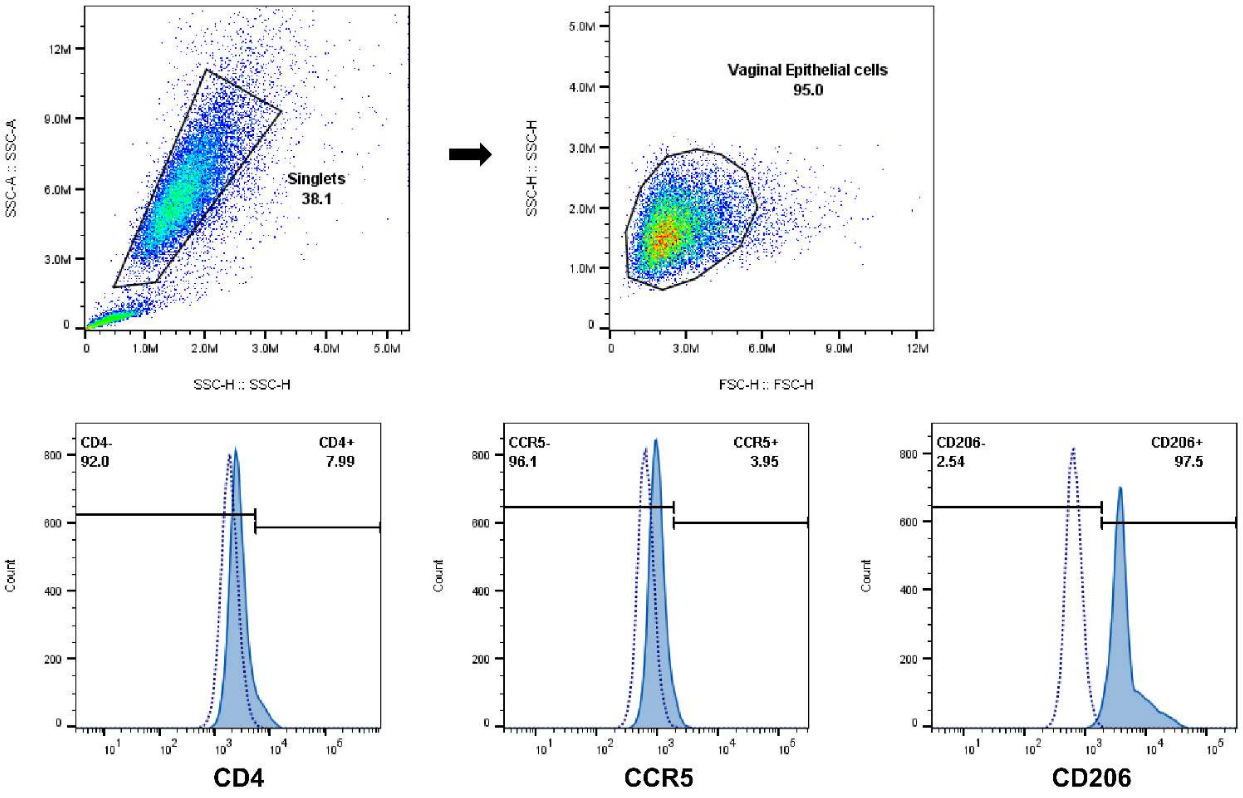

<u>Supplementary Fig.3</u> - Representative scatter plots and histograms showing gating strategy for vaginal epithelial cells in a HIV-seronegative participant. Plots were generated using FlowJo version 10.6.

Supplementary Fig.4: Expression of hMR in Vk2/E6E7

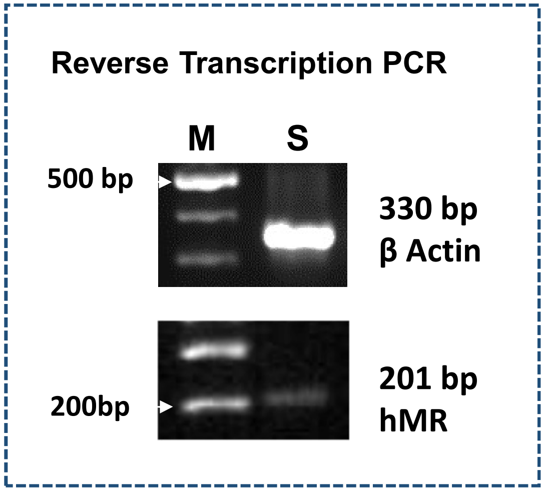

Supplementary Fig.4 - Reverse transcription PCR to detect human mannose receptor (hMR) expression (bottom panel) in Vk2/E6E7 cell line with β-actin control (top panel).

Supplementary Fig.5: Detection of Viral DNA

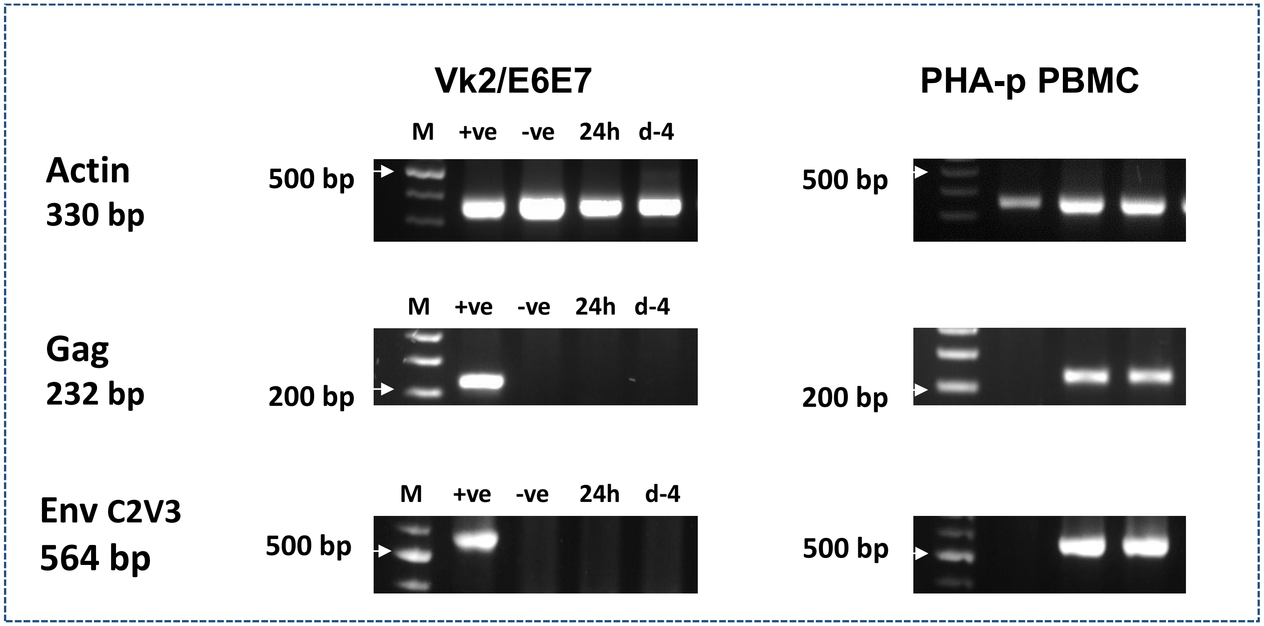

Supplementary Fig. 5 - Viral DNA detection- Absence of detectable amplification of HIV-1 gag (2nd panel) and env C2V3 (3rd panel) in Vk2/E6E7 cells at 24h and day-4 post-viral exposure, whereas distinct bands visible for PHA-p stimulated PBMCs at both time-points. β-actin control for both cell types (1st panel).

Supplementary Fig.6: Effect of HIV-1 on integrity of vaginal epithelium

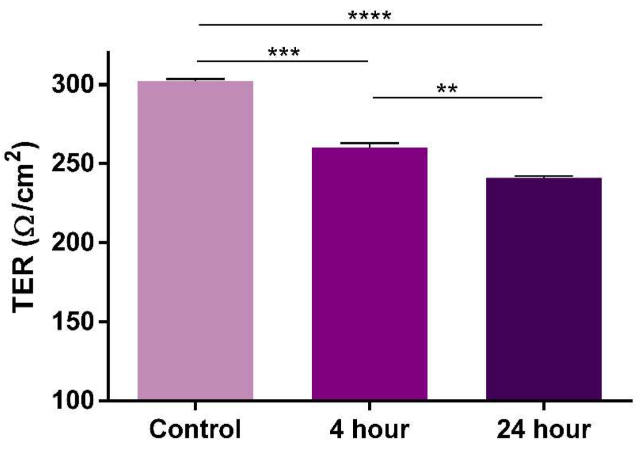

Supplementary Fig.6 - Bar graph showing significant decline in trans-epithelial resistance of Vk2/E6E7 monolayer at 4 h & 24 h post-viral exposure compared to untreated controls. Results are representative of at least three independent experiments. Statistical comparisons were made using paired t test, ** $P<0.01$, *** $P<0.001$, **** $P<0.0001$
